# Effect of physical activity on the cardiometabolic profiles of non-obese and obese subjects: Results from the Korea National Health and Nutritional Examination Survey

**DOI:** 10.1371/journal.pone.0208189

**Published:** 2019-03-01

**Authors:** Sang Joon An, Mi-Hyang Jung, Sang-Hyun Ihm, Yun-jung Yang, Ho-Joong Youn

**Affiliations:** 1 Department of Neurology, International St. Mary’s Hospital, Catholic Kwandong University College of Medicine, Incheon, Republic of Korea; 2 Cardiovascular Center, Chuncheon Sacred Heart Hospital, Hallym University College of Medicine, Chuncheon, Republic of Korea; 3 Division of Cardiology, Department of Internal Medicine, College of Medicine, The Catholic University of Korea, Seoul, Republic of Korea; 4 Institute of Biomedical Science, International St. Mary’s Hospital, Catholic Kwandong University College of Medicine, Incheon, Republic of Korea; Beijing Key Laboratory of Diabetes Prevention and Research, CHINA

## Abstract

**Background:**

Physical inactivity is an important but often neglected risk factor for various cardiovascular diseases. We hypothesized that physical inactivity might have deleterious effects on metabolic health in obese and non-obese subjects.

**Methods:**

We evaluated the effect of physical activity on the cardiometabolic profiles of a nationwide cohort of non-obese and obese individuals who did not have overt cardiovascular diseases. A total of 3,830 study subjects were divided into two groups based on their body mass index (BMI). Within each BMI group, participants were divided according to their physical activity level. To ascertain their cardiometabolic profiles, we collected data regarding the homeostasis model assessment-estimated insulin resistance (HOMA-IR) index, high-density lipoprotein (HDL)-cholesterol level, systolic blood pressure, heart rate, and high-sensitivity C-reactive protein (hsCRP) level.

**Results:**

Physically inactive subjects demonstrated markedly elevated HOMA-IR index and heart rates in each BMI category, even after adjustments for baseline covariates. They also tended to have worse profiles for HDL-cholesterol, systolic blood pressure, and hsCRP levels. A significant elevation in cardiometabolic risk was noted across the four physical activity/obesity groups (p<0.05). HOMA-IR index was largely affected by obesity, but within each BMI category, physical inactivity independently elevated the risk for worsening insulin resistance. In addition, physical inactivity significantly increased the risk of elevated heart rate in both non-obese and obese individuals. Notably, the detrimental effect of physical activity on heart rate was not modified by obesity.

**Conclusions:**

Physical activity was associated with favorable cardiometabolic risk profiles with regard to insulin resistance status and heart rate level in both BMI groups. Our results suggest that increasing physical activity could be a helpful strategy for improving the cardiometabolic health in the Korean population, regardless of obesity status.

## Introduction

Obesity is one of the top three diseases globally, with a continuously rising disease burden, according to the 2015 reports of the Global Burden of Disease Study [1]. Obesity is epidemic in Asian countries [2,3]. Furthermore, Asians are known to be more susceptible to obesity-related complications, such as insulin resistance and/or diabetes at lower body mass indices than in Western populations [2,4]. To effectively control the disease burden of obesity, an active lifestyle is needed.

Physical inactivity is an important risk factor for various cardiovascular diseases and mortality [5–12]. Despite the abundant evidence on the benefits of physical activity, most people do not engage in physical activity regularly, and this is especially common among Asians [11]. Indeed, in previous studies, only one-third of Asians were found to be physically active [13,14], and the prevalence of physical inactivity in Asia is increasing [14]. With reference to this, physicians’ indifference partly plays a role; in practice, most physicians in Asia do not counsel their patients sufficiently regarding engaging in physical activity [11,15]. Moreover, the mechanism through which physical activity confers a beneficial effect on cardiovascular health is unclear [15].

Physical activity is closely and inversely related to obesity [16,17]. Physical activity is considered one of the key factors that might explain the obesity paradox or the metabolically healthy obesity hypothesis [9]. Furthermore, even in non-obese subjects, physical inactivity might have deleterious effect on cardiometabolic health. Therefore, the effect of physical activity on the cardiometabolic health of obese and non-obese individuals needs to be clarified. However, studies in this regard are limited [18,19] and most studies focused on Western populations. Understanding the effect of physical activity on cardiometabolic profile plays an important public health role, as it could lead to appropriate counseling and the achievement of improved cardiovascular outcomes in Asians. Therefore, we conducted this study to investigate the effect of physical activity on cardiometabolic profiles, stratified by obesity, in Korean subjects without overt cardiovascular diseases, using nationwide pooled data.

## Methods

### Data source and study population

**Fig 1:**
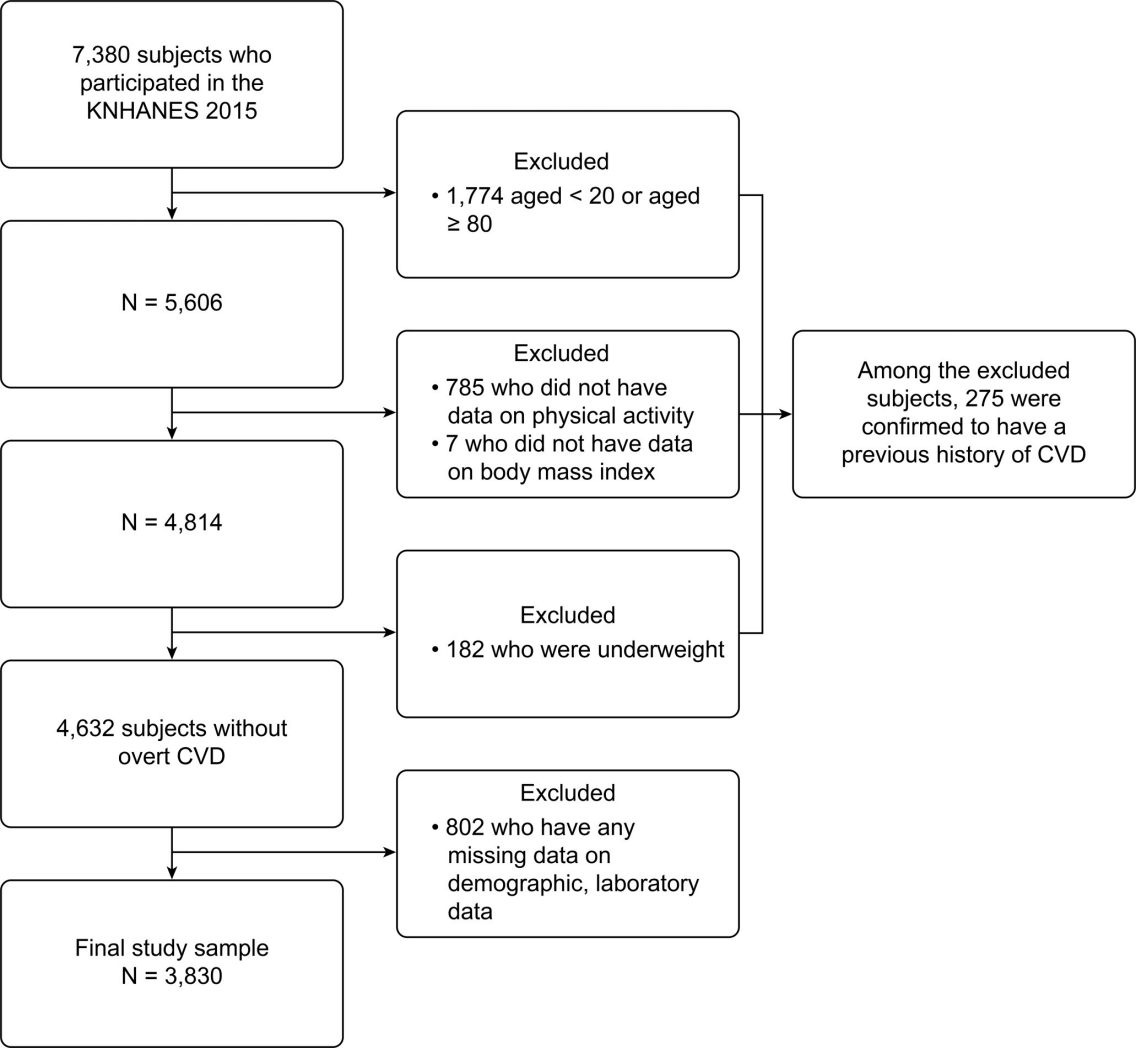
Flowchart of the study population. CVD, cardiovascular diseases; KNHANES, Korea National Health and Nutrition Examination Surveys.

Our study was based on the 2015 Korea National Health and Nutritional Examination Survey (KNHANES), which was conducted by the Korean Centers for Disease Control and Prevention (KCDC). KNHANES is a nationwide cross-sectional study, which uses a stratified multi-stage clustered probability sampling to represent the non-institutionalized civilian population of South Korea. It provides data acquired from extensive health and dietary interviews, physical examinations, and laboratory tests [20]. Among 7,380 participants in the database, we included 5,606 subjects aged 20 to 79 years. We excluded those with missing physical activity (n = 785) and body mass index (BMI; n = 7) data. We also excluded underweight participants with a BMI of less than 18.5 kg/m^2^ (n = 189) because subjects in this category may be malnourished and their characteristics may be different from those of normal-weight subjects [21,22]. Among these excluded subjects, 275 subjects were confirmed to have cardiovascular diseases (angina, myocardial infarction, stroke). We also excluded those with missing clinical and/or laboratory (n = 802) data. The final sample included 3,830 subjects. The flowchart of the study population is provided in Fig 1. All participants provided written informed consent and KCDC approved the survey protocol. All data were anonymized.

### Data collection

We collected information regarding the participants’ demographic, social/medical history, nutritional status, and laboratory findings; detailed information regarding this is provided elsewhere [20,23]. The participants’ level of education was categorized into middle school or less, high school, and college or more. Smoking status was divided into smokers (former and current) and non-smokers. Heavy drinking was defined as drinking ≥30 g/day. Hypertension status was categorized as normal blood pressure (BP<120/80 mmHg), prehypertension (120/80≤BP<140/90 mmHg), and hypertension (BP≥140/90 mmHg or the use of anti-hypertensive medications) [24]. Diabetes status was categorized as normal (fasting glucose<100 mg/dL), impaired fasting glucose (100≤fasting glucose<126 mg/dL), and diabetes (fasting glucose≥126 mg/dL, the use of antidiabetic medications/insulin, or a previous diagnosis by a physician) [25]. Daily energy intake was investigated using a 24-hour dietary recall method.

Anthropometric data were obtained using a standardized method. After ≥5 minutes of rest, BP was measured three times using a mercury sphygmomanometer by trained staff and the last two values were averaged. Heart rate was evaluated by palpating the radial pulse. When the pulse was regular, it was measured for 15 seconds and then multiplied by 4; for irregular cases, it was measured for 60 seconds.

Blood samples were collected after ≥8 hours of overnight fasting. Serum glucose, total cholesterol, triglyceride, high-density lipoprotein (HDL)-cholesterol, and low-density lipoprotein (LDL)-cholesterol levels were assessed with an automatic analyzer (Hitachi 7600–210; Hitachi High-Technologies Corporation, Tokyo, Japan). Serum insulin level was determined using a modular analyzer series (Cobas 8000; Roche, Mannheim, Germany). Homeostasis model assessment-estimated insulin resistance (HOMA-IR) index, a representative index of insulin resistance, was calculated as follows: fasting glucose (mg/dL) × fasting insulin (μIU/mL)/405 [26]. Serum high-sensitivity C-reactive protein (hsCRP) level was analyzed with Cobas 8000 using an immunoturbidimetry method.

### Assessment of obesity and physical activity

BMI was calculated as body weight divided by height squared (kg/m^2^). Obesity was defined as BMI≥25 kg/m^2^ according to the obesity criteria for the Asian population [27] and the Korean Society for the Study of Obesity [28]. We adopted this cutoff despite the differences in defining obesity in each Asia-Pacific country, because KCDC and other government organizations officially use this definition in calculating the prevalence of obesity in Korea [28,29]. The study population was categorized as non-obese (18.5≤BMI<25 kg/m^2^) or obese (BMI≥25 kg/m^2^). Physical activity was evaluated based on the Korean version of the Global Physical Activity Questionnaire [30]. According to the level of physical activity, subjects were divided into two groups: physically active and physically inactive groups. “Physically active” was defined as the performance of moderate-intensity physical activities for a minimum of 150 minutes/week, vigorous-intensity physical activities for a minimum of 75 minutes/week, or a combination of both [31].

### Study outcome: Cardiometabolic profiles

We examined the effect of physical activity on the cardiometabolic profile, which comprised three domains: 1) metabolic, 2) hemodynamic, and 3) inflammatory domains. For the metabolic domain, we evaluated HOMA-IR and lipid profiles. For the hemodynamic domain, we assessed BP and heart rate. For the inflammatory domain, hsCRP was evaluated.

### Statistical analysis

All analyses were conducted using SAS 9.4 software (SAS Institute, Cary, NC), considering the complex survey design and weighted sampling probabilities of the data source. To identify the obesity-independent role of physical activity, we stratified all subjects into two groups based on their BMI level, and within each BMI group, we divided them according to their physical activity level. Categorical variables are presented as numbers with percentages and were compared using chi-square test. Continuous variables are presented as mean ± standard error and were analyzed with Student’s *t*-test. Furthermore, analysis of covariance was performed to identify whether the difference between physically active and physically inactive subjects persisted after adjusting for baseline covariates that exhibited a significant between-group difference. The normality of the distribution was examined prior to the analysis using skewness and kurtosis. When a variable was not normally distributed, the data were log-transformed. We investigated the association between physical activity and the cardiometabolic profiles in each BMI category using linear regression analysis, treating the cardiometabolic profiles as continuous variables. Further, logistic regression analysis was conducted and the cardiometabolic profiles (study outcomes) were dichotomized using a previously known cut-off value [19,23,24,32], and we adjusted for baseline covariates to identify whether the effect of physical activity on the study outcomes was independent of other factors. A p value of <0.05 was considered statistically significant.

## Results

### Baseline characteristics of the study population

Physically active individuals were found to be younger, predominantly male, more educated, and with a lower prevalence of hypertension and diabetes compared to physically inactive subjects in each BMI category. Interestingly, there were more heavy drinkers and a higher energy intake in the physically active group than in the physically inactive group (Table 1). The distributions of the cardiometabolic profiles are provided in S1 Table.

**Table 1:** Baseline characteristics of the study population (n = 3,830).

Variable	Non-obese			Obese		
	PA	PI	p	PA	PI	p
**Number**	611	1,852		309	1,058	
**Clinical variable**						
Age, years	41.1 ± 0.7	47.3 ± 0.5	<0.0001	42.4 ± 0.8	50.9 ± 0.6	<0.0001
Male sex	279 (45.7)	689 (37.2)	0.0005	201 (65.1)	456 (43.1)	<0.0001
Body mass index, kg/m^2^	22.2 ± 0.1	22.2 ± 0.1	0.5467	27.6 ± 0.2	27.8 ± 0.1	0.4162
Education			<0.0001			<0.0001
≤Middle school	82 (13.4)	640 (34.6)		63 (20.4)	485 (45.8)	
High school	248 (40.6)	606 (32.7)		106 (34.3)	320 (30.3)	
≥College	281 (46.0)	606 (32.7)		140 (45.3)	253 (23.9)	
Smoking	247 (40.4)	601 (32.5)	0.0524	165 (53.4)	434 (41.0)	0.0550
Heavy drinking	314 (51.4)	747 (40.3)	0.0005	183 (59.2)	466 (44.1)	<0.0001
Energy intake, cal/day	2346.5 ± 57.0	2050.0 ± 26.9	<0.0001	2411.3 ± 73.0	2150.6 ± 42.2	0.0002
Hypertension status			<0.0001			0.0003
Normal BP	376 (61.5)	939 (50.7)		106 (34.3)	288(27.2)	
Prehypertension	129 (21.1)	410 (22.1)		98 (31.7)	252 (23.8)	
Hypertension	106 (17.4)	503 (27.2)		105 (34.0)	518 (49.0)	
Diabetes status			<0.0001			<0.0001
Normal	467 (76.4)	1,268 (68.5)		189 (61.2)	508 (48.0)	
IFG	113 (18.5)	366 (19.8)		87 (28.2)	379 (35.8)	
Diabetes	31 (5.1)	218 (11.8)		33 (10.7)	171 (16.2)	
**Hemodynamic profile**						
Systolic BP, mmHg	112.0 ± 0.6	115.1 ± 0.5	<0.0001	120.7 ± 0.9	123.3 ± 0.6	0.0166
Diastolic BP, mmHg	73.2 ± 0.4	73.6 ± 0.3	0.4657	78.8 ± 0.6	78.6 ± 0.4	0.8416
Heart rate, bpm	67.6 ± 0.5	70.1 ± 0.3	<0.0001	68.7 ± 0.7	69.9 ± 0.4	0.1128
**Metabolic profile**						
Fasting glucose, mg/dL	93.3 ± 0.6	98.1 ± 0.6	<0.0001	99.7 ± 1.2	105.4 ± 0.9	0.0003
HOMA-IR	1.43 ± 0.05	1.59 ± 0.03	0.003	2.53 ± 0.09	3.16 ± 0.11	<0.0001
Total cholesterol, mg/dL	187.6 ± 1.7	189.5 ± 0.9	0.3086	196.6 ± 1.9	197.8 ± 1.4	0.6056
Triglyceride, mg/dL	108.4 ± 4.3	120.4 ± 2.7	0.0165	180.3 ± 11.6	177.6 ± 5.6	0.8289
HDL-cholesterol, mg/dL	54.7 ± 0.6	53.3 ± 0.3	0.0408	46.2 ± 0.7	46.3 ± 0.4	0.8289
LDL-cholesterol, mg/dL	111.5 ± 1.5	112.7 ± 0.8	0.5042	121.7 ± 1.9	120.8 ± 1.3	0.6988
**Inflammatory profile**						
hsCRP, mg/dL	0.94 ± 0.07	1.09 ± 0.07	0.1140	1.38 ± 0.12	1.67 ± 0.08	0.0413

Data are presented as mean ± standard error or numbers (percentages). BP, blood pressure; HDL, high-density lipoprotein; HOMA-IR, homeostasis model assessment-estimated insulin resistance; hsCRP, high-sensitivity C-reactive protein; IFG, impaired fasting glucose; LDL, low-density lipoprotein; PA, physically active; PI, physically inactive.

### Different cardiometabolic profiles based on physical activity

**Fig 2:**
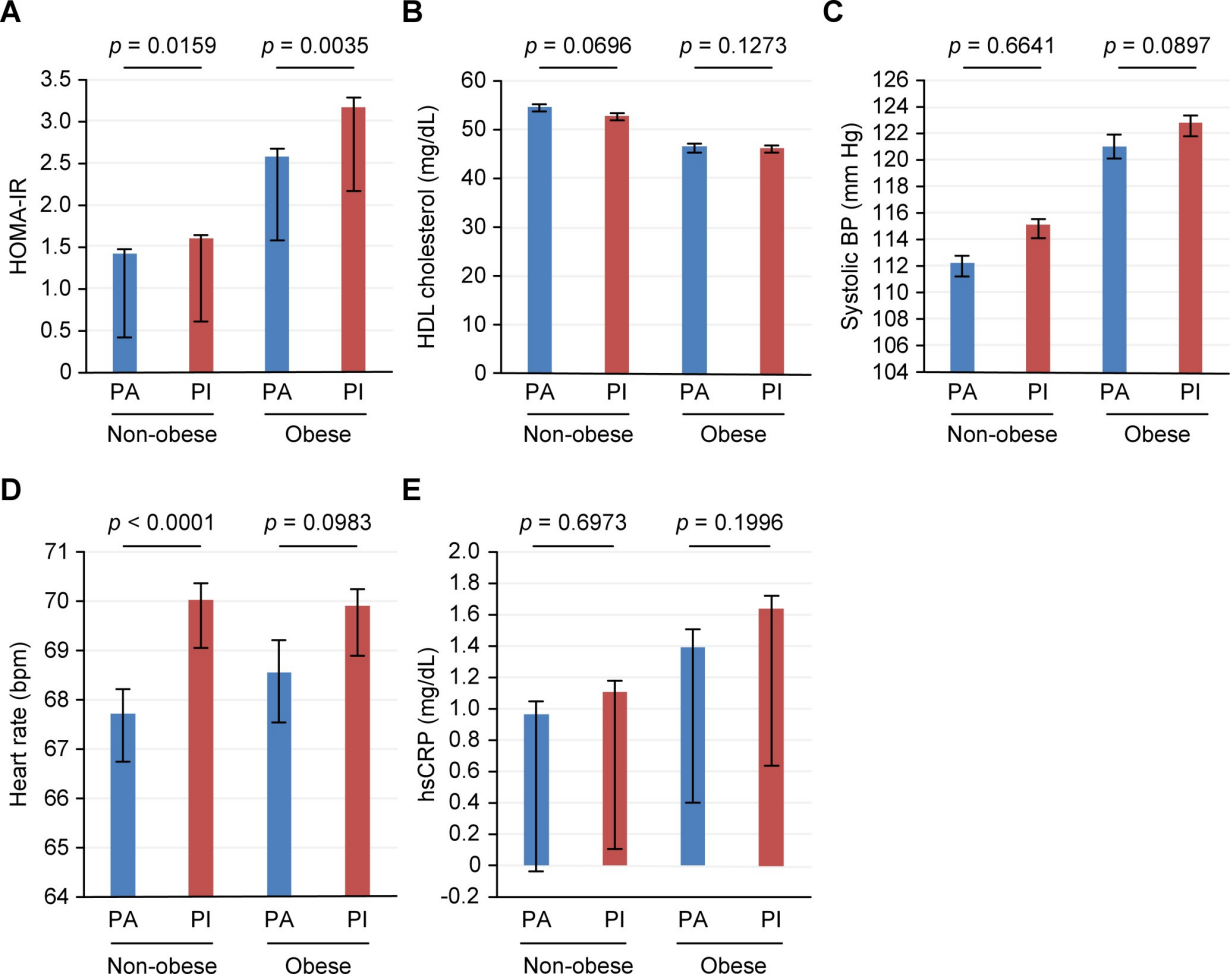
Differences in the cardiometabolic profiles of physically active and physically inactive subjects in both BMI groups. The blue bars indicate physically active subjects, and the red bars denote physically inactive subjects. BP, blood pressure; HDL, high-density lipoprotein; HOMA-IR, homeostasis model assessment-estimated insulin resistance; hsCRP, high-sensitivity C-reactive protein.The error bars represent the adjusted mean ± standard deviation. Data are adjusted for age, sex, education level, alcohol consumption, energy intake, and hypertension and diabetes statuses.

HOMA-IR was significantly lower in physically active subjects than in physically inactive subjects in each BMI category. This significance remained after adjustments for baseline demographic confounders, such as age, sex, education level, alcohol consumption, energy intake, hypertension, and diabetes. Heart rate was lower in the physically active subjects, and this difference was more statistically significant in the non-obese group. HDL-cholesterol level was higher, and systolic BP and hsCRP levels were lower in physically active subjects than in physically inactive subjects; although the statistical significance was lost after controlling for confounders (Fig 2).

### Association between physical activity and cardiometabolic profile

Physical inactivity was positively associated with HOMA-IR and heart rate in both non-obese and obese subjects. Physical inactivity was negatively related to HDL-cholesterol level and positively associated with hsCRP level, although these associations were not statistically significant (Table 2).

**Table 2:** Multivariate linear regression analysis evaluating the association between physical inactivity and the cardiometabolic profiles in each body mass index category.

	Non-obese			Obese		
	β-coefficient	Standard error	p	β-coefficient	Standard error	p
**Metabolic profile**						
HOMA-IR	0.121	0.052	0.0224	0.471	0.153	0.0024
HDL-cholesterol, mg/dL	-1.107	0.630	0.0808	-1.207	0.790	0.1284
**Hemodynamic profile**						
Systolic BP, mmHg	-0.229	0.434	0.5987	-1.335	0.830	0.1099
Heart rate, bpm	2.423	0.526	<0.0001	1.385	0.743	0.0641
**Inflammatory profile**						
hsCRP, mg/dL	0.018	0.091	0.8446	0.198	0.154	0.2014

Standardized regression coefficients (β-coefficient) are adjusted for age, sex, education level, alcohol consumption, energy intake, and hypertension and diabetes statuses. BP, blood pressure; bpm, beats per minute; HDL, high-density lipoprotein; HOMA-IR, homeostasis model assessment-estimated insulin resistance; hsCRP, high-sensitivity C-reactive protein.

**Fig 3:**
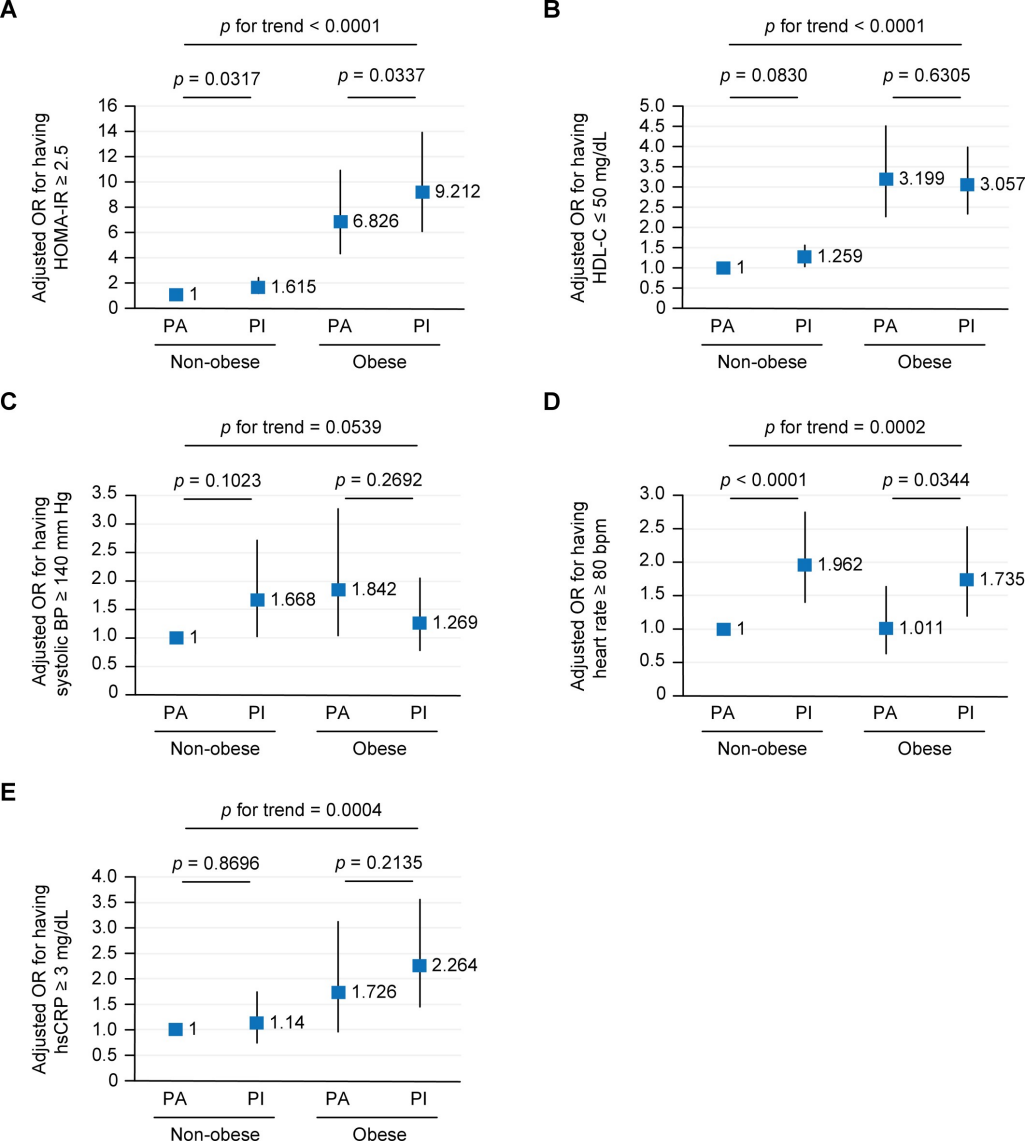
Effect of physical activity on cardiometabolic profile in non-obese and obese individuals. A significant elevation in cardiometabolic risk was noted across the 4 physical activity/obesity groups (p for trend <0.05). (A) HOMA-IR was largely affected by obesity, but within each BMI category, physical inactivity independently elevated the risk for worsening insulin resistance. (B) Similar to HOMA-IR, HDL-C was largely affected by obesity. Physical inactivity marginally elevated the risk for a lower HDL-C level in the non-obese group, whereas no such significant risk elevation was observed in the obese group. This might indicate that the obese group had a more progressed metabolic disturbance state to effectively intervene with physical activity than the non-obese group. (C) Physical activity did not show any significant risk-benefit for systolic BP elevation in either BMI group. (D) Regarding heart rate, physical inactivity significantly elevated the risk for an elevated heart rate in both non-obese and obese individuals; notably, the detrimental effect of physical activity on heart rate was not modified by obesity. (E) Physical inactivity elevated the risk for an unfavorable inflammatory state in both BMI groups, although this finding was not statistically significant. BP, blood pressure; HDL-C, high-density lipoprotein-cholesterol; HOMA-IR, homeostasis model assessment-estimated insulin resistance; hsCRP, high-sensitivity C-reactive protein; OR, odds ratio The data were adjusted for age, sex, education level, alcohol consumption, energy intake, and hypertension and diabetes statuses.

Further, we evaluated the association between physical activity and cardiometabolic profiles using logistic regression analysis. Specifically, we assessed the effect of physical inactivity on HOMA-IR in subjects with or without obesity (Fig 3A). In both non-obese and obese individuals, physical inactivity significantly increased the risk for HOMA-IR ≥2.5, even after controlling for age, sex, education level, alcohol consumption, energy intake, and hypertension and diabetes statuses (p<0.05). The detrimental effect of physical inactivity was additional to that of obesity and appeared to be more profound among obese subjects. Physically inactive subjects with obesity had the highest risk for HOMA-IR ≥2.5 (odds ratio [OR] 9.121; 95% confidence interval [CI] 6.095–13.912), followed by physically active subjects with obesity (OR 6.826; 95% CI 4.271–10.909), and non-obese physically inactive subjects (OR 1.615; 95% CI 1.104–2.363), compared to the reference group of non-obese physically active subjects. Regarding heart rate, physical inactivity significantly elevated the risk for a heart rate of ≥80 bpm in both non-obese and obese individuals (p <0.05, Fig 3D). Interestingly, the detrimental effect of physical activity on heart rate was not modified by obesity. Physically active subjects exhibited a similar risk for an elevated heart rate regardless of their obesity status. Further, non-obese but physically inactive subjects had a greater risk for an elevated heart rate (OR 1.962; 95% CI 1.397–2.754) compared to obese but physically active subjects (OR 1.011; 95% CI 0.625–1.633). Other profiles, such as HDL-cholesterol, systolic BP, and hsCRP revealed inconsistent or insignificant associations with physical activity (Fig 3).

## Discussion

In an apparently healthy representative Korean adult sample, we observed the beneficial effect of physical activity on cardiometabolic profile in two BMI groups. The key findings are as follows: 1) physical activity was significantly associated with a lower insulin resistance and heart rate in both BMI groups, 2) physical activity was associated with nonsignificant but better HDL-cholesterol, systolic BP, and hsCRP levels, and 3) physical inactivity elevated the risk for insulin resistance and increased heart rate in both non-obese and obese subjects, independent of baseline confounders. Further, we observed an independent effect of physical activity on heart rate, which was unmodified by obesity; however, with regard to insulin resistance, obesity was the major contributor to insulin resistance and further physical activity independently affected insulin resistance. Collectively, our data suggest that promoting physical activity could be a useful strategy for improving cardiometabolic health in the Korean population, regardless of obesity status, in those at high risk for developing metabolic diseases at a given BMI.

### Beneficial role of physical activity in insulin resistance

The current study is in line with previous studies that demonstrated the beneficial effect of physical activity/fitness on insulin resistance or glucose homeostasis [18,33,34]. Chen et al. previously reported that a higher level of physical activity could improve glucose tolerance and pancreatic ß-cell function, independent of obesity in a community dwelling of 1,152 Mexican Americans [33]. It is noteworthy that self-directed physical activity, without a separate exercise intervention, could improve glucose metabolism, which was corroborated in our study. In a recent study, which included 1,247 European children, Nystrőm et al. observed that obesity was associated with an unfavorable insulin resistance profile and that physical fitness significantly counteracted insulin resistance, especially in obese children. Contrary to our results, the protective effect of physical fitness was not noted in normal-weight children. This might be attributable to the difference in the ages of the study population; unlike children, adults would have some degree of metabolic derangement, although they are normal-weight, and physical activity might be more helpful in reversing the derangement. Thus, our data support the notion that physical activity should be encouraged regardless of obesity status. Although the underlying mechanism by which physical activity confers a beneficial effect on insulin resistance is not fully understood, physical activity has been known to markedly increase glucose uptake in the muscle and liver, thereby, reducing serum glucose level [35]. Further, physical activity may enhance insulin sensitivity by reducing adipose tissue [36,37], which could block the vicious cycle of obesity-driven insulin resistance [36,38], decrease inflammatory cytokines, and increase adiponectin release from adipose tissues [33,39].

### Beneficial effect of physical activity on heart rate

We observed an independent effect of physical activity on heart rate, which was unmodified by obesity; however, with regard to insulin resistance, obesity was the major contributor to insulin resistance, and further physical activity independently affected insulin resistance. Our data indicate that physical activity and obesity are independent determinants of insulin resistance, while physical activity, is an independent determinant of an elevated heart rate. These findings are in line with those of previous reports that demonstrated an inverse relationship between an elevated heart rate and physical activity/fitness, and indicated that heart rate is a marker of physical activity [40–42]. In a recent prospective study, Nauman et al. reported that after 23 years of follow-up, subjects with an elevated heart rate (≥80 bpm) at baseline were associated with a decreased physical fitness level compared to those with a lower heart rate (<60 bpm) at baseline [40]. Presumably, physical activity is associated with a lower heart rate, which in turn results in a better fitness level, leading to a virtuous cycle. Further, a lower heart rate could offer sufficient time for diastolic left ventricular filling and coronary flow and improve arterial compliance. Moreover, improvements in autonomic nervous system function, reflected by a lower heart rate, could exert a beneficial effect on cardiometabolic mortality, directly or indirectly, by mediating an improvement in metabolic and/or inflammatory system function, all of which are closely interrelated [41,43–45]. However, the precise mechanism by which physical activity lowers heart rate remains unclear. Corroborating our data, previous studies reported that exercise training lowers heart rate (training-induced bradycardia) [42–48]. Physical activity/exercise increases parasympathetic activity and decreases sympathetic activity via cardiovascular adaptation to exercise, with a net effect of lowering heart rate [46,47]. This neural control works through multiple levels including central command, baroreceptor reflex, and neural reflex feedback from the muscle [47]. Further, physical activity possibly affects the autonomic system by enhancing the sympathoinhibitory effect of nitric oxide [49].

### Implications of the current study

Our data has clinical and public health implications. Considering the increasing disease burden and different metabolic characteristics of obesity (risk for insulin resistance at a given BMI) in the Asian population, it is crucial to encourage more people to engage in physical activity. Physical activity, as a feasible approach from a public health perspective, could improve insulin resistance and heart rate level. Recent studies revealed that engaging in only 5 to 10 minutes of exercise daily could effectively reduce all-cause and cardiovascular mortality [10,11,50], although the most effective mode and frequency of exercise need to be clarified. It is noteworthy that the beneficial effect of physical activity was noted in both the non-obese and obese subjects. In the clinical setting, physicians more actively tend to counsel their obese patients to engage in physical activity; however, non-obese patients are often not counselled similarly, based on the assumption that they are in a metabolically healthy condition. Our data suggest that non-obese individuals also need counseling regarding the promotion of their cardiometabolic health. For research purposes, our data provide additional evidence of the mechanistic role of physical activity in the prevention of cardiometabolic diseases.

### Strengths and limitations of our study

This study was based on a representative Korean sample and is one of the largest studies assessing the association between physical activity and various cardiometabolic profiles in an Asian population. Further, we controlled for multiple demographic confounders, including education level, comorbidities, and energy intake. Notably, physically active subjects exhibited lower HOMA-IR levels and heart rate, although they had a higher energy intake. This finding highlights the importance of maintaining physical activity regardless of energy intake and other demographic characteristics.

Our study had some limitations. First, the cross-sectional design of the study precluded the identification of causal associations. Second, the definition of obesity, as used in this study, might be inappropriate for other ethnicities or other Asian countries. We acknowledge that heterogeneity exists in defining obesity in each Asia-Pacific country [51]. Third, assessing physical activity based on a self-reported questionnaire was an indirect method compared to other methods, such as the treadmill test and the cardiopulmonary exercise test. However, it enabled the evaluation of physical activity on a population level and further provided evidence that participating in self-directed physical activity could effectively improve a person’s cardiometabolic profile.

## Conclusions

In a healthy Korean population without overt cardiovascular diseases, physical activity was associated with a better insulin resistance and heart rate profile. These beneficial effects of physical activity were shown in both non-obese and obese populations. Our results provide evidence for the need to promote physical activity in order to improve cardiometabolic outcomes in Asians.

## Supporting information

S1 Table Data distribution of cardiometabolic profiles assessed by skewness and kurtosis. (DOCX)
