# Supplementary material for: Effect of physical activity on the cardiometabolic profiles of non-obese and obese subjects: Results from the Korea National Health and Nutritional Examination Survey
Source: PLoS One. 2019 Mar 1;14(3):e0208189. doi: 10.1371/journal.pone.0208189 (PMC6396903; doi:10.1371/journal.pone.0208189)
Supplement: S1 Table — (DOCX) [file pone.0208189.s001.docx]

S1 Table. Data distribution of cardiometabolic profiles assessed by skewness and kurtosis

| Variable | Normal weight / PA | | Normal weight / PI | | Obese / PA | | Obese / PI | |
| --- | --- | --- | --- | --- | --- | --- | --- | --- |
|  | Skewness | Kurtosis | Skewness | Kurtosis | Skewness | Kurtosis | Skewness | Kurtosis |
| Systolic BP, mmHg | 1.205 | 5.763 | 0.796 | 3.688 | 0.896 | 4.516 | 0.544 | 3.759 |
| Heart rate, bpm | 0.549 | 3.313 | 0.547 | 3.604 | 5.314 | 60.936 | 0.567 | 4.589 |
| HOMA-IR | 3.134 | 21.594 | 3.510 | 25.264 | 5.364 | 52.660 | 7.421 | 87.159 |
| HDL-cholesterol, mg/dL | 0.455 | 3.389 | 0.685 | 4.096 | 0.603 | 3.468 | 0.887 | 4.481 |
| hsCRP, mg/dL | 5.434 | 40.070 | 5.366 | 36.419 | 4.163 | 25.006 | 4.345 | 25.843 |

BP, blood pressure; HDL, high-density lipoprotein; HOMA-IR, homeostasis model assessment-estimated insulin resistance; hsCRP, high-sensitivity C-reactive protein; PA, physically active; PI, physically inactive
